# Protective Effect of Sex Hormone-Binding Globulin against Metabolic Syndrome: *In Vitro* Evidence Showing Anti-Inflammatory and Lipolytic Effects on Adipocytes and Macrophages

**DOI:** 10.1155/2018/3062319

**Published:** 2018-06-25

**Authors:** Hiroki Yamazaki, Akifumi Kushiyama, Hideyuki Sakoda, Midori Fujishiro, Takeshi Yamamotoya, Yusuke Nakatsu, Takako Kikuchi, Sunao Kaneko, Hirotoshi Tanaka, Tomoichiro Asano

**Affiliations:** ^1^Department of Rheumatology and Allergy, IMSUT Hospital, The Institute of Medical Science, The University of Tokyo, 4-6-1 Shirokanedai, Minato-ku, Tokyo 108-8639, Japan; ^2^Division of Diabetes and Metabolism, Institute for Adult Diseases, Asahi Life Foundation, 2-2-6, Bakuro-cho, Chuo-ku, Tokyo 103-0002, Japan; ^3^Division of Neurology, Respirology, Endocrinology and Metabolism, Department of Internal Medicine, Faculty of Medicine, University of Miyazaki, 5200 Kihara, Kiyotake-cho, Miyazaki, Miyazaki 889-1692, Japan; ^4^Division of Diabetes and Metabolic Diseases, Nihon University School of Medicine, 30-1, Oyaguchi Kami-cho, Itabashi-ku, Tokyo 173-8610, Japan; ^5^Department of Medical Science, Graduate School of Medicine, University of Hiroshima, 1-2-3 Kasumi, Minami-ku, Hiroshima, Hiroshima 734-8551, Japan; ^6^Department of Diabetes and Metabolic Diseases, The University of Tokyo Hospital, 7-3-1 Hongo, Bunkyo-ku, Tokyo 113-8655, Japan

## Abstract

Sex hormone-binding globulin (SHBG) is a serum protein released mainly by the liver, and a low serum level correlates with a risk for metabolic syndrome including diabetes, obesity, and cardiovascular events. However, the underlying molecular mechanism(s) linking SHBG and metabolic syndrome remains unknown. In this study, using adipocytes and macrophages, we focused on the *in vitro* effects of SHBG on inflammation as well as lipid metabolism. Incubation with 20 nM SHBG markedly suppressed lipopolysaccharide- (LPS-) induced inflammatory cytokines, such as MCP-1, TNF*α*, and IL-6 in adipocytes and macrophages, along with phosphorylations of JNK and ERK. Anti-inflammatory effects were also observed in 3T3-L1 adipocytes cocultured with LPS-stimulated macrophages. In addition, SHBG treatment for 18 hrs or longer significantly induced the lipid degradation of differentiated 3T3-L1 cells, with alterations in its corresponding gene and protein levels. Notably, these effects of SHBG were not altered by coaddition of large amounts of testosterone or estradiol. In conclusion, SHBG suppresses inflammation and lipid accumulation in macrophages and adipocytes, which might be among the mechanisms underlying the protective effect of SHBG, that is, its actions which reduce the incidence of metabolic syndrome.

## 1. Introduction

SHBG is a 40–50 kDa protein mainly synthesized in the liver and secreted into the bloodstream. This protein is comprised of two laminin G-like (LG) domains [1], and the molecular weights of serum SHBG proteins are partially dependent on their glycosylation status [2]. The conventional roles of SHBG involve transporting sex hormones and the regulation of hormone dynamics [3]. Numerous studies have confirmed the relationship between the serum SHBG concentration and metabolic syndrome. Low SHBG concentrations correlate with higher levels of serum inflammatory markers [4, 5]. Relatively low levels of SHBG are also a risk factor for obesity, metabolic syndrome, and diabetes [6–10]. Thus, the serum SHBG concentration has been regarded as a biomarker for metabolic syndrome.

On the other hand, interestingly, db/db mice overexpressing human SHBG reportedly show resistance to the development of obesity and hepatosteatosis [11, 12]. In addition, a single nucleotide polymorphism related to an elevated plasma SHBG concentration reportedly correlates with a reduced risk of diabetes [13]. The hormone-like effect of SHBG has also been demonstrated in experiments using prostatic cells [14, 15], MCF-7 breast cancer cells [16], cytotrophoblasts [17], proximal tubule epithelial cells [18, 19], and hepatocytes [12]. The results of these previous studies led us to speculate that the serum SHBG level is not only simply a consequence of altered metabolic conditions but also exerts favorable effects protecting against the development of metabolic disorders.

In this study, using adipocytes and macrophages, we focused on the *in vitro* effects of SHBG in inflammation as well as lipid metabolism, since lipid accumulation and inflammation are both necessary for the development of metabolic syndrome. Herein, we present evidence of the favorable actions of SHBG in adipocytes and macrophages.

## 2. Materials and Methods

### 2.1. Chemicals and Reagents

SHBG protein was purchased from two companies, Abcam (ab151275) and Fitzgerald Industries (30-AS40). According to the explanations provided by these manufacturers, SHBG protein was purified from human sera, and its purity exceeded 90%. While we confirmed the effects of SHBG from these two companies to be the same, the data presented herein were those obtained with the SHBG from Abcam. In addition, we measured the amounts of testosterone and estradiol contaminating the SHBG protein, since no information was given regarding this issue in the materials provided by the manufacturers. Sex hormones were measured using the ELISA kits for testosterone and estradiol (Cayman) according to the manufacturer's instructions. Diethyl ether was added to the SHBG protein samples and mixed thoroughly with a vortex. The upper ether layer was collected using a pasteur pipette and transferred into a clean tube. This extraction procedure was repeated four times. After evaporating the combined ether extracts, the samples were dissolved in the buffer and subjected to analysis with the ELISA kits.

Lipopolysaccharide (LPS) (from *Escherichia coli* 0111:B4) was purchased from Sigma. Recombinant murine TNF*α* was purchased from Genzyme (3410T). Anti-*β*-actin antibody (sc-1616), anti-CCAAT/enhancer binding protein *α* (CEBP*α*) antibody (sc-61), and horseradish peroxidase- (HRP-) labeled anti-goat IgG antibody (sc-2020) were from Santa Cruz Biotechnology. Anti-TNF*α* antibody (#11948), anti-stress-activated c-Jun amino-terminal kinase (JNK)1/2 antibody (#9252), anti-phospho-extracellular signal-related kinase (ERK)1/2 (Thr202/Tyr204) antibody (#9101), anti-ERK1/2 antibody (#9102), anti-adipose triglyceride lipase (ATGL) antibody (#2439), HRP-labeled anti-rabbit (#7074), and anti-mouse IgG antibody (#7076) were all from Cell Signaling Technology. Anti-phospho-JNK1/2 (Thr183/Tyr185) antibody was obtained from BD Biosciences (#562480).

### 2.2. 3T3-L1 Cell Culture and Differentiation

3T3-L1 cells were differentiated as previously described [20, 21] with some modifications. Briefly, 3T3-L1 cells were cultured in Dulbecco's Modified Eagle's medium (DMEM) (Wako) containing 10% donor calf serum (Invitrogen) in a 5–10% CO_2_ incubator. For the experiments, cells were spread onto collagen type I coated plates (Iwaki) and induced to differentiate with DMEM containing 10% fetal calf serum (FCS) (Biowest), 0.5 mM 3-isobutyl-1-methylxanthine (Sigma), 4 *μ*g/ml dexamethasone (Sigma), and 167 nM insulin (Sigma). Two days later, the media were replaced with DMEM containing 10% FCS and 167 nM insulin. After another two days, the media were replaced with DMEM containing 10% FCS and the media were then changed every other day. Penicillin-streptomycin (Invitrogen) was added to all media at a 0.5% concentration.

For experiments on mature 3T3-L1 cells, we used cells that had been differentiating for more than 6 days [22]. Mature adipocytes were treated with SHBG proteins in phenol red-free DMEM (Wako) containing 0.2% fatty acid-free bovine serum albumin (BSA) (Wako). The concentration of BSA was much higher than that of SHBG protein. We used phenol red-free media to eliminate estrogen-like effects of phenol red [23].

For experiments evaluating inflammatory cytokine levels in adipocytes, 3T3-L1 cells were pretreated overnight with 20 nM SHBG, followed by stimulation with 1 ng/ml LPS or 1 ng/ml TNF*α* for 12–24 hrs. In some experiments, 1 or 20 *μ*M testosterone (T) (Wako, 208-08341) or 17*β*-estradiol (E_2_) (Sigma, E8875) was coadded with 0–20 nM of SHBG protein. Considering the amounts and the reported association constants of T and E_2_ with SHBG (1.6 × 10^9^ M^−1^ and 6.8 × 10^8^ M^−1^, resp. [24]), it was assumed that more than 99% of SHBG would form a complex with T or E_2_.

### 2.3. Quantitative Reverse-Transcription Polymerase Chain Reaction (qRT-PCR)

Total RNA was extracted using the RNeasy Mini Kit (Qiagen). Reverse transcription was performed using Transcriptor Universal cDNA Master (Roche) followed by RT-PCR employing LightCycler 480 SYBR Green I Master (Roche). Sequences of the primers used in this study are listed in Table 1. The 36B4 mRNA level served as the internal control.

### 2.4. Preparation of Mouse Peritoneal Macrophages

The isolation protocol was reported previously [39, 40] and achieved a final cell population comprised of more than 90% macrophages. We employed this protocol, with a slight modification. Peritoneal macrophages were collected from C57BL/6N mice. RPMI 1640 media without phenol red (Gibco) was used, and the cells were incubated at 37°C in a 5% CO_2_ incubator. Macrophages were collected by injection of 5 ml of RPMI 1640 media containing 10% FCS intraperitoneally under diethyl ether anesthesia and then left on ice until centrifugation. After centrifugation at 1500 rpm for 2 minutes at room temperature, the supernatant was removed, and hemolysis buffer (BD PharmLyse) was added to remove the red blood cells. After 2 minutes, we centrifuged the samples at 1500 rpm for 2 minutes at room temperature and the supernatant was removed. Cells were seeded at a density of 1.5 × 10^6^ cells/well in a 12-well plate in RPMI 1640 media containing 10% FCS. Two hours later, the cells were gently washed twice with RPMI 1640 media to remove nonadherent cells and the medium was then replaced with RPMI 1640 containing 0.2% fatty acid-free BSA for the experiments. For experiments evaluating inflammatory cytokine levels in macrophages, cells were pretreated with 20 nM SHBG overnight, followed by 1 ng/ml LPS for 0–8 hrs.

### 2.5. Immunoblotting Analysis

The cells were solubilized with Laemmli buffer (0.2 M Tris·HCl, 4% SDS, 10% glycerol, 5% 2-mercaptoethanol, and 0.1% bromophenol blue) containing 100 mM dithiothreitol. Equal amounts of protein from whole cell lysates were resolved by SDS-PAGE. Then, the proteins were transferred to Immobilon (Millipore), blocked with 1% BSA (Intergen), reacted with the primary antibodies and subsequently with the HRP-labeled secondary antibodies. Chemiluminescence was detected using an ImageQuant LAS 4000 mini (Fuji Film).

### 2.6. Coculture System of 3T3-L1 Adipocytes and Murine Macrophages

The 3T3-L1 adipocytes and murine macrophages were cocultured in a transwell system (Corning, Acton, MA) with a 0.4 *μ*m porous membrane to separate the upper and lower chambers. Mouse peritoneal macrophages were harvested and seeded at a density of 1.5 × 10^6^ cells/well in the upper chamber, while differentiated 3T3-L1 cells were in the lower chamber. Both macrophages and differentiated 3T3-L1 cells were washed with RPMI 1640 medium, and the culture medium was then replaced with RPMI 1640 containing 0.2% fatty acid-free BSA for the experiments. SHBG at the 20 nM concentration was added to both the upper and the lower chambers, and coculture was then started. After incubation overnight, 100 pg/ml LPS was added and the cells were collected 12 hrs later.

### 2.7. Lipid Staining

Differentiated 3T3-L1 cells were treated with 0–100 nM SHBG protein in serum-free media and incubated for 3 days, followed by Oil Red O staining or Nile Red staining.

Oil Red O (Sigma) was dissolved in isopropanol to assure that the concentration would be 0.3%. This stock solution was mixed with distilled water (3 : 2), followed by incubation for 30 minutes, and filtered with 0.45 *μ*m before use. The cells were washed twice with PBS and fixed with 10% neutral buffered formalin for 10 minutes. After the cells were washed twice with PBS, Oil Red O working solution was added followed by another 10-minute incubation and then washed with PBS. Images were taken using a light microscope FSX100 (Olympus).

Nile Red (AdipoRed, Lonza) becomes fluorescent when it is partitioned in a hydrophobic environment and shows selective fluorescence for intracellular lipid droplets [41]. The staining protocol was carried out according to the manufacturer's instructions. Fluorescence with excitation at 485 nm and emission at 535 nM was measured using ARVO MX-fla (PerkinElmer).

### 2.8. Measurement of Glycerol in the Culture Medium

Differentiated 3T3-L1 cells were treated with 20 nM SHBG protein for 18 or 35 hrs. Glycerol concentrations in culture media were measured employing a Glycerol Assay Kit (Sigma). With this kit, the glycerol concentration is determined by a coupled enzyme assay involving glycerol kinase and glycerol phosphate oxidase, resulting in a colorimetric product.

### 2.9. cAMP Measurement

Differentiated 3T3-L1 cells were treated with 20 nM SHBG protein or 10 *μ*M isoproterenol for 1 or 18 hrs. Intracellular cAMP concentrations were measured using a cAMP EIA kit (Cayman). This assay is based on the competition between free cAMP and a cAMP tracer. Measurements were carried out according to the manufacturer's instructions. Stimulation with isoproterenol (Sigma) was used to confirm the production of cAMP.

### 2.10. Data Analysis

All data are presented as the means ± standard deviation (S.D.). The differences between two groups were evaluated by *t*-test. *p* < 0.05 was considered to indicate a statistically significant difference.

## 3. Results

### 3.1. SHBG Suppressed LPS- or TNF*α*-Induced Inflammatory Cytokine Levels in Mouse Peritoneal Macrophages and Differentiated 3T3-L1 Cells

First of all, we measured the concentrations of testosterone and 17*β*-estradiol contaminating the SHBG protein purchased from Abcam, to exclude the possibility of its functions being attributable to these sex hormones. The results obtained with the ELISA assay kits revealed that Abcam's SHBG protein contained molar ratios of 1 : 5600 and 1 : 10000 for testosterone and estradiol, respectively, to SHBG. Therefore, it is unlikely that the contaminant testosterone and 17*β*-estradiol contributed to the results obtained using Abcam's SHBG protein in this study.

**Figure 1:**
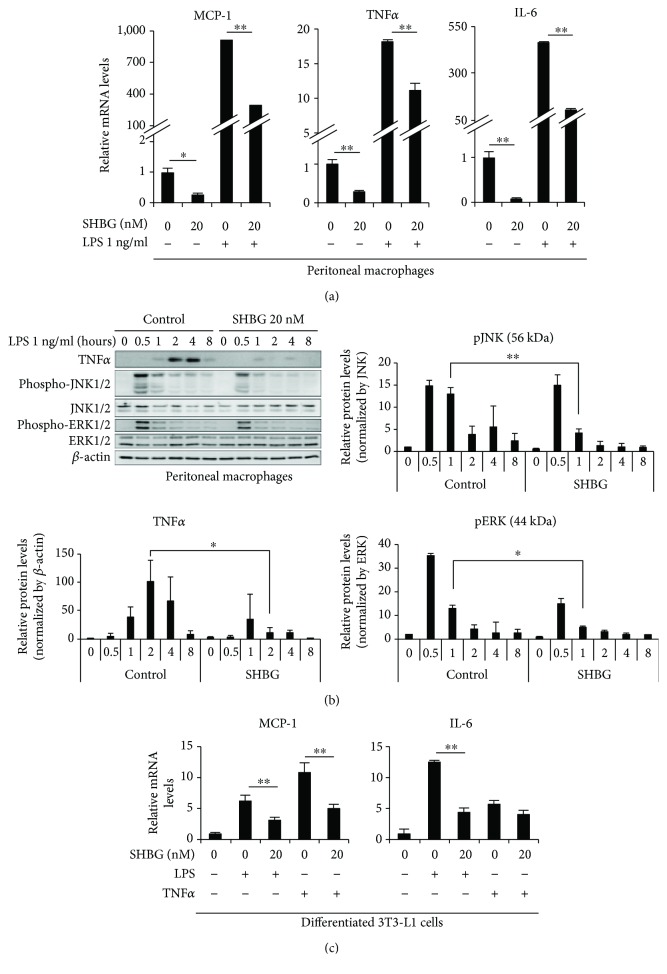
SHBG inhibits inflammatory cytokine levels in peritoneal macrophages and differentiated 3T3-L1 cells. (a) Peritoneal macrophages from C57BL/6 mice were treated with SHBG overnight, followed by 1 ng/ml LPS stimulation for 8 hrs. mRNA levels of inflammatory cytokines were measured by RT-PCR. Student's *t*-test was performed. Data are the means ± S.D. (*n* = 4, ^∗^*p* < 0.05, ^∗∗^*p* < 0.01). (b) Peritoneal macrophages from C57BL/6 mice were treated with SHBG protein overnight, followed by 1 ng/ml LPS stimulation for the indicated times. Inflammatory signaling was evaluated by Western blotting. Each band was quantified using ImageJ. Relative intensities are shown. Data are the means ± S.D. (*n* = 3, ^∗^*p* < 0.05, ^∗∗^*p* < 0.01). (c) Differentiated 3T3-L1 cells were treated with SHBG proteins overnight, followed by 1 ng/ml LPS or 1 ng/ml TNF*α* stimulation for 24 hrs. mRNA levels of MCP-1 and IL-6 were measured by RT-PCR. Student's *t*-test was performed. Data are the means ± S.D. (*n* = 3, ^∗∗^*p* < 0.01).

Murine macrophages were stimulated with or without 1 ng/ml LPS for 8 hrs, and the effects of 20 nM SHBG were examined. While LPS markedly raised mRNA levels of monocyte chemoattractant protein-1 (MCP-1), TNF*α*, and IL-6, SHBG suppressed them under both basal and LPS-stimulated conditions in the approximate range of 50–90% (Figure 1(a)).

Next, the effects of SHBG on the signal transductions leading to inflammatory cytokine levels were investigated. Maximal phosphorylations of JNK1/2 and ERK1/2 occurred at 30 min after the addition of 1 ng/ml LPS, while the intracellular TNF*α* content peaked around 2 hrs. In the presence of 20 nM SHBG, LPS-induced phosphorylations of JNK1/2 and ERK1/2 as well as TNF*α* production were reduced. The band intensity of TNF*α* normalized by *β*-actin at 2 hrs, phosphorylation of JNK normalized by JNK at 1 hr, and phosphorylation of ERK normalized by ERK at 1 hr were significantly decreased (Figure 1(b)).

Similarly, the effects of SHBG on MCP-1 and IL-6 levels in 3T3-L1 adipocytes were investigated, by stimulating these cells with LPS or TNF*α* and comparing the results to those in adipocytes without stimulation. It was revealed that 20 nM SHBG markedly suppressed LPS-induced MCP-1 and IL-6 mRNA upregulations as well as TNF*α*-induced MCP-1 levels (Figure 1(c)). These results indicate that SHBG exerts anti-inflammatory effects directly on macrophages and adipocytes.

### 3.2. Inflammatory Cytokine Levels Were Also Suppressed in the Coculture System of Peritoneal Macrophages and 3T3-L1 Adipocytes

**Figure 2:**
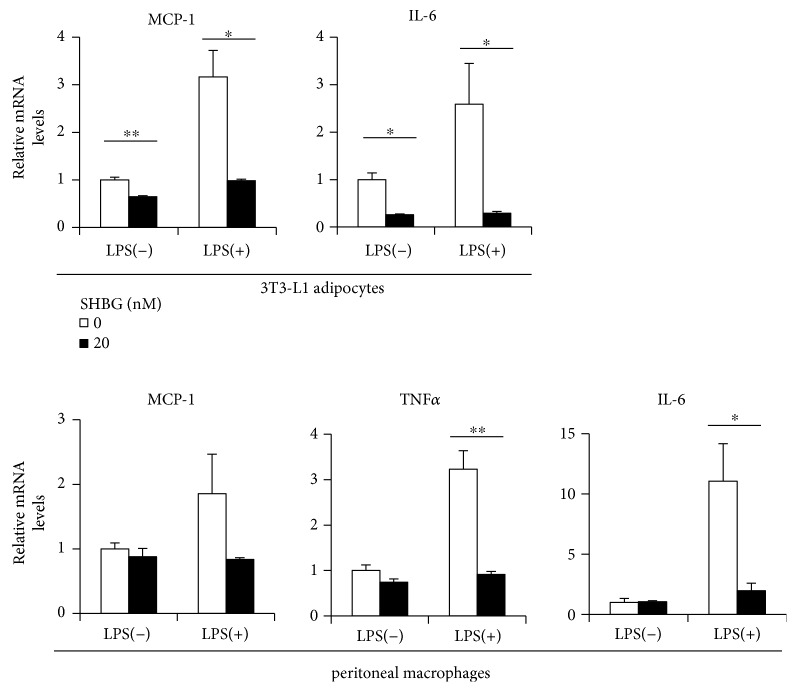
SHBG inhibits inflammatory cytokine levels in 3T3-L1 cells cocultured with peritoneal macrophages. Differentiated 3T3-L1 cells and peritoneal macrophages from C57BL/6 were cocultured using a transwell system overnight, in culture media with or without SHBG protein. Thereafter, we added 100 pg/ml LPS to the culture media and cells were collected 12 hrs later. mRNA levels of inflammatory cytokines in each cell were measured by RT-PCR. Student's *t*-test was performed. Data are the means ± S.D. (*n* = 3, ^∗^*p* < 0.05, ^∗∗^*p* < 0.01).

We cocultured 3T3-L1 adipocytes and murine macrophages using a transwell system. Then, LPS was added, and the resulting cytokine levels in both 3T3-L1 adipocytes and macrophages were compared between the presence and the absence of 20 nM SHBG. In this experiment, SHBG exerted inhibitory effects on basal cytokine levels in 3T3-L1 adipocytes. Notably, the addition of 20 nM SHBG markedly suppressed LPS-induced MCP-1 and IL-6 levels in 3T3-L1 adipocytes (Figure 2(a)), as well as MCP-1, TNF*α*, and IL-6 levels in murine macrophages (Figure 2(b)). Although the optimal medium for 3T3-L1 cells is DMEM, coculturing in RPMI did not apparently impair the functions of 3T3-L1 cells.

### 3.3. SHBG Reduced the Lipid Accumulation in 3T3-L1 Adipocytes

**Figure 3:**
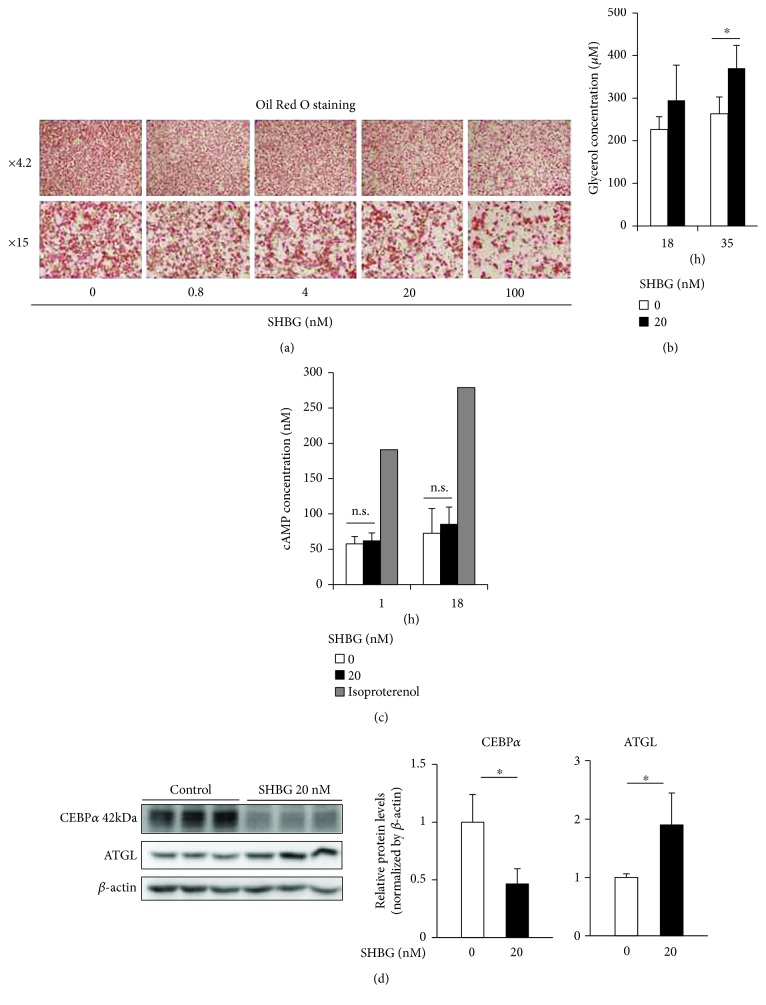
SHBG reduces lipid contents of differentiated 3T3-L1 cells with alterations in corresponding protein levels. (a) Differentiated 3T3-L1 cells were treated with SHBG proteins at the indicated concentrations in serum-free media and incubated for 3 days. Oil Red O staining was performed. Representative fluorescent microscopy images are shown. (b) Differentiated 3T3-L1 cells were treated with 20 nM SHBG proteins for 18 or 35 hrs. Glycerol concentrations in culture media were measured by ELISA. Student's *t*-test was performed. Data are the means ± S.D. (*n* = 3, ^∗^*p* < 0.05). (c) Differentiated 3T3-L1 cells were treated with 20 nM SHBG proteins or 10 *μ*M isoproterenol for 1 or 18 hrs. Intracellular cAMP concentrations were measured. Student's *t*-test was performed. Data are the means ± S.D. (SHBG 0 and 20 nM: *n* = 4). (d) Differentiated 3T3-L1 cells were treated with 20 nM SHBG proteins for 3 days. Protein levels of CEBP*α* and ATGL were evaluated by Western blotting. Each band was quantified using ImageJ. Relative intensities normalized by *β*-actin are shown. Data are means ± S.D. (*n* = 3, ^∗^*p* < 0.05).

Differentiated 3T3-L1 cells were treated with SHBG proteins at the indicated concentrations in serum-free media and incubated for 3 days and followed by Oil Red O staining (Figure 3(a)). It was revealed that SHBG protein reduced lipid accumulation in 3T3-L1 adipocytes in a concentration-dependent manner. Glycerol concentrations in the culture media were increased in the presence of 20 nM SHBG for 18 or 35 hrs (Figure 3(b)), which suggests lipolysis to be enhanced by SHBG. It was found that SHBG did not alter the intracellular cAMP concentration, in contrast to the marked cAMP increase induced by isoproterenol (Figure 3(c)). Interestingly, treatment with 20 nM SHBG proteins for 3 days markedly reduced CEBP*α* and increased ATGL proteins (Figure 3(d)).

### 3.4. SHBG Altered the mRNA Levels Related to Lipid Metabolism in Differentiated 3T3-L1 Adipocytes

**Figure 4:**
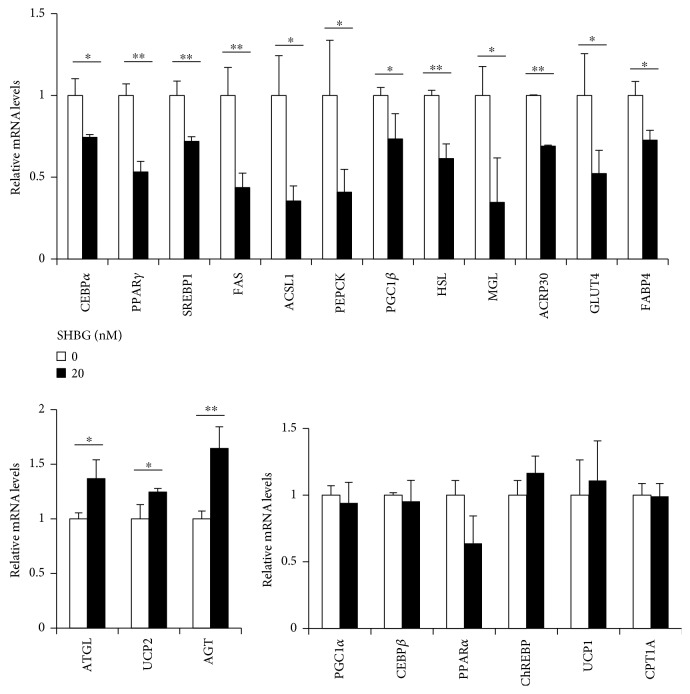
SHBG alters some of the mRNA levels related to lipid metabolism in differentiated 3T3-L1 cells. Differentiated 3T3-L1 cells were treated with 20 nM SHBG proteins for 18 hrs. mRNA levels were measured by RT-PCR. Relative levels normalized by the 36B4 level are shown. Student's *t*-test was performed. Data are the means ± S.D. (*n* = 3, ^∗^*p* < 0.05, ^∗∗^*p* < 0.01).

Differentiated 3T3-L1 cells were treated with 20 nM SHBG protein for 18 hrs, and mRNA levels were measured by RT-PCR. Importantly, mRNA levels of CEBP*α*, peroxisome proliferator-activated receptor *γ* (PPAR*γ*), and sterol regulatory element-binding protein 1 (SREBP1), gene encoding key transcriptional factors for adipogenic differentiation and triglyceride synthesis, are significantly downregulated by 18 hrs of incubation with 20 nM SHBG. The genes downregulated by SHBG included fatty acid synthase (FAS), acyl-CoA synthetase 1 (ACSL1), phosphoenolpyruvate carboxykinase (PEPCK), PPAR*γ* co-activator-1*β* (PGC1*β*), hormone-sensitive lipase (HSL), monoacylglycerol lipase (MGL), adipocyte complement-related protein of 30 kDa (ACRP30), glucose transporter type 4 (GLUT4), and fatty acid binding protein 4 (FABP4), while ATGL, uncoupling protein-2 (UCP2), and angiotensinogen (AGT) were all upregulated. PGC1*α*, CEBP*β*, PPAR*α*, carbohydrate response element binding protein (ChREBP), UCP1, and carnitine palmitoyltransferase 1A (CPT1A) were not significantly changed. Taken together, these observations raise the possibility that SHBG induces dedifferentiation via downregulation of its key transcription factors and lipid metabolism genes (Figure 4).

### 3.5. Coincubations with an Excess of Testosterone or 17*β*-Estradiol Did Not Affect the Function of SHBG

**Figure 5:**
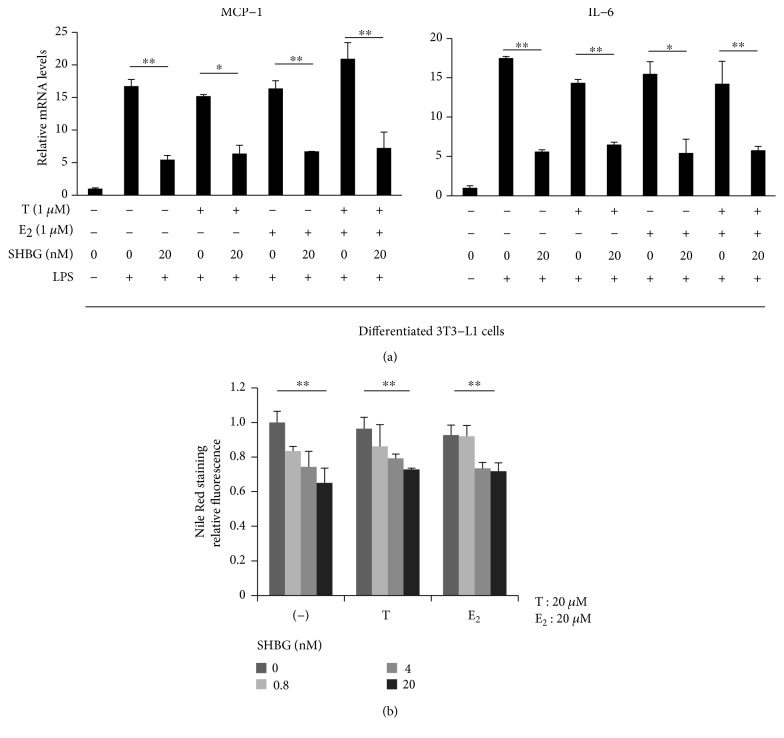
Coincubation with testosterone or 17*β*-estradiol did not affect the function of SHBG. (a) Differentiated 3T3-L1 cells were treated with SHBG protein in the presence or absence of 1 *μ*M testosterone (T) or 17*β*-estradiol (E_2_) overnight, followed by stimulation with 1 ng/ml LPS for 12 hrs. mRNA levels of MCP-1 and IL-6 were measured by RT-PCR. Student's *t*-test was performed. Mean ± S.D. (*n* = 3, ^∗^*p* < 0.05, ^∗∗^*p* < 0.01). (b) Differentiated 3T3-L1 cells were treated with SHBG protein at concentrations ranging from 0–20 nM in serum-free media containing 20 *μ*M of testosterone (T) or 17*β*-estradiol (E_2_). Three days later, Nile Red staining was performed and fluorescence was quantified. The Jonckheere test was performed. Mean ± S.D. (*n* = 4, ^∗∗^*p* < 0.01).

Differentiated 3T3-L1 cells were treated with 20 nM SHBG in the presence or absence of 1 *μ*M testosterone or 17*β*-estradiol overnight and then stimulated with 1 ng/ml LPS for 12 hrs. The suppressive effects of SHBG on MCP-1 and IL-6 levels were unaffected by testosterone or 17*β*-estradiol (Figure 5(a)). Similarly, no significant effect of testosterone or 17*β*-estradiol on the reduced lipid content by SHBG was observed in 3T3-L1 adipocytes (Figure 5(b)).

## 4. Discussion

In the present study, it was clearly demonstrated that SHBG exhibits anti-inflammatory effects involving macrophages and adipocytes, as evidenced by suppressed mRNA levels for inflammatory cytokines such as MCP-1, TNF*α*, and IL-6. MCP-1, which is known to be highly expressed in adipocytes, is related to the induction of chronic inflammation [42]. Chronic inflammation in adipose tissues is reportedly exacerbated by LPS from the intestinal tract accompanied by obesity or high-fat diets [43–45]. In addition, it is very likely that SHBG enhances lipolysis or induces dedifferentiation of mature adipocytes, based on the effects on a series of mRNA level data. Under conditions of obesity, macrophages reportedly infiltrate adipose tissue, and interactions between macrophages and adipocytes occur via a paracrine mechanism [46], which exacerbates the metabolic syndrome phenotype. Our experiments using a coculture system yielded results supporting the anti-inflammatory effects of SHBG.

It should be noted that the SHBG concentration used in this study is physiological. The median serum SHBG concentration is 20.8 nM in young adult men, increasing to 44.5 nM with aging [47]. Women have serum SHBG concentrations several times higher than those in men, reaching approximately 100 nM [47, 48]. Thus, the 20 nM mainly used in our experiments is the approximate normal lower limit. Thus, it may be reasonable to regard SHBG as contributing to protection from metabolic syndrome accompanying inflammation and obesity.

Assuming the presence of a specific receptor for SHBG, signal transduction from the SHBG receptor reportedly suppresses the phosphorylations of JNK and ERK, possibly inhibiting the activation of transcriptional factors such as AP-1 [49, 50]. AP-1 regulates MCP-1, a key chemokine for monocyte/macrophage migration and infiltration [51]. Lipolytic actions of SHBG were observed to be accompanied by changes in various mRNA and protein levels. Key transcription factors such as CEBP*α*, PPAR*γ*, and SREBP1 controlling adipogenesis and lipogenesis were suppressed by SHBG. SHBG might influence the metabolic processes in adipocytes by modulating nutrient usage or hormonal cascades including growth factor signaling. There are many other documented mechanisms of action of SHBG. The increased intracellular cAMP levels in several cells [14, 16, 17] suggest the involvement of G protein and adenylate cyclase, though neither of these responses was observed in our present experiments. SHBG protein itself might not exert activity inducing signaling cascades. For example, SHBG reportedly competes with osteocalcin-induced signaling by binding to GPRC6A [52]. Such chronic and low-grade inhibition or modulation of other protein receptor-mediated processes might be important. Although SHBG is certainly a trace protein in serum, the local concentrations in tissues can be high, considering the finding that the fibulin family sequesters SHBG and possibly controls access of some molecules to target cells [53, 54]. Furthermore, the internalization of SHBG and actions within cells, reportedly enhancing or inhibiting sex hormone actions [15, 18, 19], might be physiologically meaningful. The modes of SHBG action might differ depending on the targeted cell or phenotype, although the relevant SHBG receptor(s) has not yet been identified. Further investigations are necessary to unravel these mechanisms.

SHBG exists as a complex with sex hormones to some degree in sera. In human sera, the proportion of unbound SHBG to total SHBG is 50% in men and 80% in women [55]. Considering the reported association constants [24], the coincubations with an excess of testosterone or 17*β*-estradiol in our experiments were postulated to have saturated the binding sights of SHBG proteins. One limitation of this study is that we could not determine precisely the proportions of SHBG protein coupled and uncoupled with sex steroids when excess amounts of sex hormones were added. However, considering that very small amounts of sex steroids were present as contaminants of Abcam's SHBG protein and that the addition of excess amounts of sex steroids did not affect the actions of SHBG, it is reasonable to regard SHBG as exerting anti-inflammatory and lipolytic actions regardless of whether or not it is coupled with sex hormones.

It is possible that the actions of SHBG observed herein might be modified by residual steroids in cells, which had proliferated in serum-containing media before the experiments. Sex hormones exert effects on certain cell types via SHBG and the putative SHBG receptor complex, as previously reported [56, 57].

In rodents, the Shbg gene is not expressed in the liver postnatally. The role of SHBG in rodents might be limited during the fetal period. However, our findings suggest that human SHBG protein exerts activity on adipocytes and macrophages derived from mice. These findings are concordant with the report that human SHBG-Tg mice with the db/db background are resistant to the development of obesity [11]. Effects of human SHBG protein on human adipocytes or macrophages warrant further examinations.

SHBG concentration changes have previously been considered to result from metabolic abnormalities including inflammation [58] and hepatic lipogenesis [59]. Thus, SHBG may be regarded as a good biomarker for metabolic syndrome. However, our results also raise the possibility that SHBG suppresses chronic inflammation, in good agreement with several previous studies employing SHBG transgenic mice, and also exerts direct effects on numerous cell types, as mentioned in the Introduction.

## 5. Conclusions

In conclusion, at a physiological concentration, SHBG suppresses inflammation and lipid accumulation in macrophages and adipocytes, which may be among the mechanisms underlying the protective effect of SHBG which acts to suppress the development of metabolic syndrome.

## Figures and Tables

**Table 1:** Primer sequences used for this study.

*Gene*	Forward primer (5′ → 3′)	Reverse primer (5′ → 3′)	Reference
*36B4*	GCTCCAAGCAGATGCAGCA	CCGGATGTGAGGCAGCAG	[25]
*Mcp-1*	AGGTCCCTGTCATGCTTCTG	TCTGGACCCATTCCTTCTTG	[26]
*Tnfa*	GAACTGGCAGAAGAGGCACT	AGGGTCTGGGCCATAGAACT	[26]
*Il-6*	TCGTGGAAATGAGAAAAGAGTTG	AGTGCATCATCGTTGTTCATACA	[27]
*Cebpa*	TGAGCCGTGAACTGGACACG	CAGCCTAGAGATCCAGCGAC	[28]
*Pparg*	TCTTCCATCACGGAGAGGTC	GATGCACTGCCTATGAGCAC	[28]
*Srebp1*	AAGCAAATCACTGAAGGACCTGG	AAAGACAAGGGGCTACTCTGGGAG	[29]
*Fas*	ATCCTGGAACGAGAACACGATCT	AGAGACGTGTCACTCCTGGACTT	[30]
*Acsl1*	GACGACCTCAAGGTGCTTCA	ACCCAGGCTCGACTGTATCT	—
*Pepck*	CTAACTTGGCCATGATGAACC	CTTCACTGAGGTGCCAGGAG	[31]
*Pgc1b*	GCTCTGACGCTCTGAAGGAC	CACCGAAGTGAGGTGCTTATG	[30]
*Hsl*	CAGTGCCTATTCAGGGACAGAG	CACTCCTGCGCATAGACTCC	—
*Mgl*	AGGCGAACTCCACAGAATGTT	AGCCAGCTCATCATAACGGC	—
*Acrp30*	GCTCCTGCTTTGGTCCCTCCAC	GCCCTTCAGCTCCTGTCATTCC	[32]
*Glut4*	CAGCTCTCAGGCATCAAT	TCTACTAAGAGCACCGAG	[33]
*Fabp4*	TGGGAACCTGGAAGCTTGTC	CTTTCCTTGTGGCAAAGCCC	—
*Atgl*	AACACCAGCATCCAGTTCAA	GGTTCAGTAGGCCATTCCTC	[34]
*Ucp2*	CTACAAGACCATTGCACGAGAGG	AGCTGCTCATAGGTGACAAACAT	[35]
*Agt*	AGGTTGGCGCTGAAGGATAC	GATGTATACGCGGTCCCCAG	[36]
*Pgc1a*	GCCCGGTACAGTGAGTGTTC	CTGGGCCGTTTAGTCTTCCT	[30]
*Cebpb*	CAAGCTGAGCGACGAGTACA	AGCTGCTCCACCTTCTTCTG	[37]
*Ppara*	CGGGAAAGACCAGCAACAAC	TGGCAGCAGTGGAAGAATCG	[30]
*Chrebp*	GATGGTGCGAACAGCTCTTCT	CTGGGCTGTGTCATGGTGAA	[30]
*Ucp1*	GATGGTGAACCCGACAACTT	CTGAAACTCCGGCTGAGAAG	[38]
*Cpt1a*	GACTCCGCTCGCTCATTCC	ACCAGTGATGATGCCATTCTTG	—
